# The normal-weight metabolic syndrome: Clinical manifestations and pathogenic mechanisms

**DOI:** 10.1016/j.metop.2026.100513

**Published:** 2026-10-27

**Authors:** John Nzobokela, Bisom Chikwanka Mweene, Elletina Nchimunya Sinamwenda, Situmbeko Liweleya, Sepiso K. Masenga, Lweendo Muchaili, Benson M. Hamooya

**Affiliations:** a Department of Cardiovascular Science and Metabolic Diseases, Livingstone Center for Prevention and Translational Sciences, Livingstone, Zambia; b Nursing Department, School of Nursing and Midwifery, Mulungushi University, Kabwe, Zambia; c Pathology and Microbiology Department, School of Medicine and Health Sciences, Mulungushi University, Livingstone, Zambia; d Department of Public Health, School of Medicine and Health Sciences, Mulungushi University, Livingstone, Zambia; e Department of Medicine, Vanderbilt University Medical Center, Nashville, TN, USA; f Vanderbilt Center for Immunobiology, Vanderbilt Institute for Infection, Immunology and Inflammation, Vanderbilt University Medical Center, Nashville, TN, USA; g Vanderbilt Institute for Global Health, Vanderbilt University Medical Center, Nashville, TN, USA

## Abstract

Metabolic syndrome (MetS), conventionally associated with obesity, also occurs in individuals with a normal body mass index (BMI), revealing an important discordance between body weight and metabolic health. This phenotype, variably described as normal-weight metabolic syndrome (NWMS), metabolically obese normal weight (MONW), or metabolically unhealthy normal weight (MUNW), is characterized by metabolic abnormalities despite the absence of generalized obesity. Reported prevalence varies substantially across populations because of heterogeneity in phenotype definitions, BMI thresholds, metabolic criteria, and population characteristics, and the global burden remains uncertain. Nevertheless, affected individuals have an increased risk of type 2 diabetes and cardiovascular disease compared with metabolically healthy normal-weight individuals. This review critically synthesizes current evidence on the epidemiology, clinical manifestations, diagnostic approaches, and pathogenic mechanisms of NWMS. Visceral and ectopic adiposity, insulin resistance, adipose tissue dysfunction, and chronic low-grade inflammation represent the most consistently supported pathogenic pathways. In contrast, gut microbiota dysbiosis, oxidative stress, ferroptosis, and related molecular processes remain emerging mechanisms with limited evidence specific to normal-weight populations. Recognition of metabolic risk beyond BMI is therefore important, particularly in susceptible populations. A risk-based approach incorporating conventional metabolic assessment, measures of central adiposity, and selective body-composition assessment may improve identification of affected individuals. Harmonized phenotype definitions and studies across geographically and ethnically diverse populations are needed to define the global burden and inform targeted prevention and management strategies.

## Graphical abstract

**Figure:**
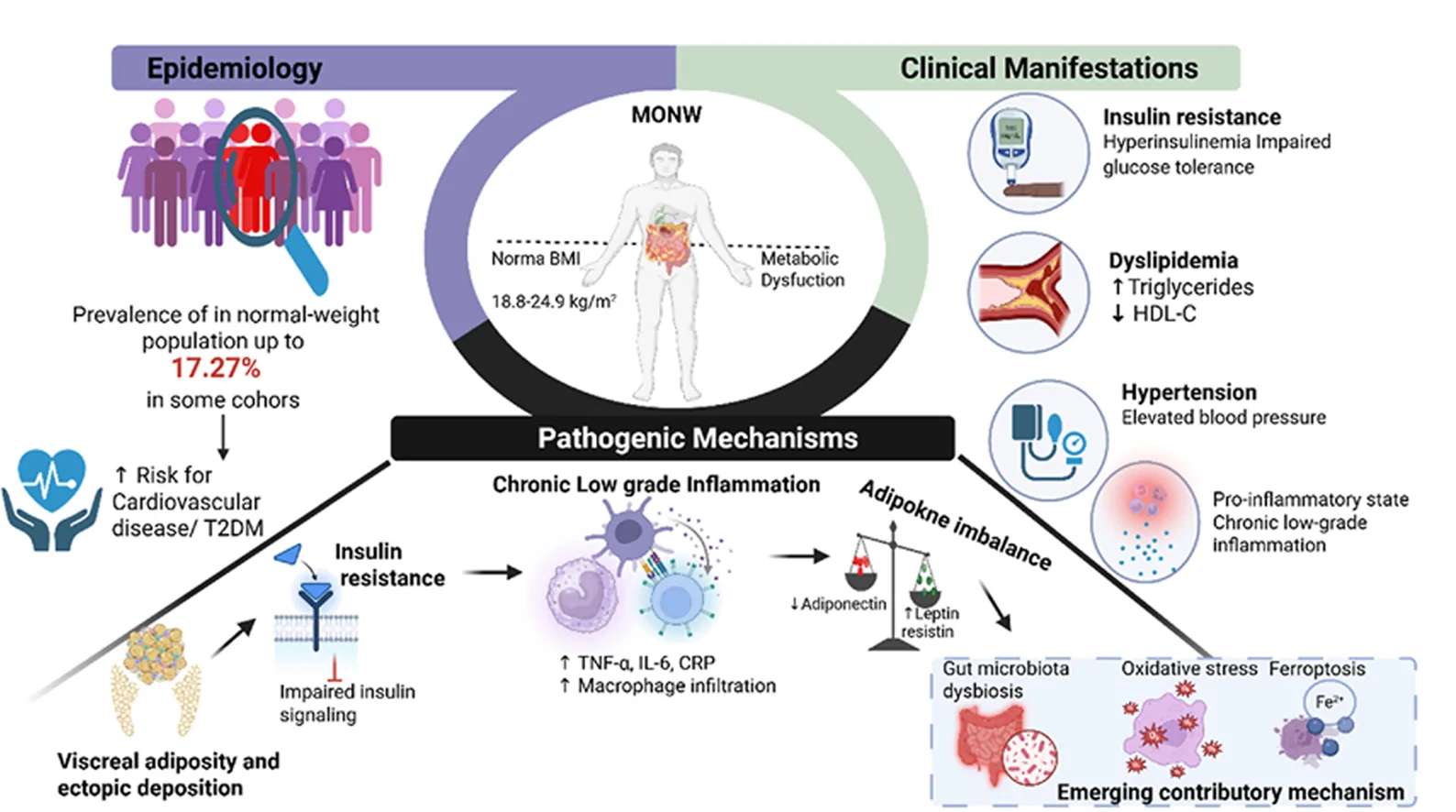

## 1 Introduction

Metabolic syndrome (MetS) is characterized by the clustering of dysglycemia, hypertension, atherogenic dyslipidemia, and central adiposity and is associated with increased risks of type 2 diabetes mellitus and cardiovascular disease [1], [2]. Although these abnormalities are strongly associated with excess adiposity, body mass index (BMI) does not reliably distinguish metabolic health from metabolic disease. A clinically important subset of individuals with normal BMI exhibits insulin resistance and other cardiometabolic abnormalities typically associated with obesity. This discordance between body weight and metabolic health challenges BMI-centered risk assessment and highlights the importance of body-fat distribution, ectopic adiposity, and adipose tissue function.

The phenotype was first conceptualized by Ruderman and colleagues as the “metabolically obese, normal-weight” (MONW) individual [3]. Subsequent studies have used several related terms that describe overlapping but distinct phenotypes. Metabolically unhealthy normal weight (MUNW) broadly refers to normal-weight individuals with metabolic abnormalities, although definitions vary across studies, whereas normal-weight metabolic syndrome (NWMS) refers more specifically to individuals with normal BMI who meet established diagnostic criteria for MetS. Normal-weight obesity (NWO) differs conceptually because it is defined primarily by excessive body-fat percentage despite normal BMI and does not necessarily indicate metabolic dysfunction [4], [5], [6]. In this review, NWMS is used when established MetS criteria are fulfilled, MONW/MUNW when referring to studies using broader definitions of metabolic dysfunction, and NWO when excess body fat is the defining characteristic. This distinction is important because heterogeneity in phenotype definitions, BMI thresholds, and metabolic criteria contributes substantially to variation in prevalence estimates and limits direct comparison across studies[5], [6], [7].

The clinical importance of metabolic dysfunction at normal weight extends beyond its prevalence. Compared with metabolically healthy normal-weight individuals, affected individuals have increased risks of type 2 diabetes, cardiovascular disease, and premature mortality[8], [9], [10]. Metabolic abnormalities may also coexist with conditions such as metabolic dysfunction-associated steatotic liver disease and chronic kidney disease, reinforcing the systemic nature of the phenotype [11], [12]. Importantly, metabolic risk is heterogeneous even within the normal BMI range and may vary according to age, sex, ethnicity, body-fat distribution, and genetic and environmental susceptibility[13], [14], [15]. These observations underscore the limitations of using BMI alone to identify individuals at cardiometabolic risk.

Despite increasing recognition of NWMS and related phenotypes, important uncertainties remain. Reported prevalence varies substantially across populations because of inconsistent definitions and differences in ethnicity, metabolic thresholds, and body-composition assessment, while evidence from several regions, particularly Africa, remains limited [7], [13], [16]. Mechanistically, visceral and ectopic fat accumulation, insulin resistance, adipose tissue dysfunction, and chronic low-grade inflammation are supported by a substantial body of evidence, whereas the contributions of gut microbiota dysbiosis, oxidative stress, ferroptosis, and genetic and epigenetic factors remain less well established specifically in normal-weight populations [17], [18]. Moreover, much of the mechanistic and therapeutic evidence has been extrapolated from obesity-associated metabolic disease, making it important to distinguish established mechanisms from emerging hypotheses in NWMS.

Therefore, this review critically synthesizes current evidence on the epidemiology, clinical manifestations, diagnostic approaches, and pathogenic mechanisms of metabolic syndrome in normal-weight individuals. Particular emphasis is placed on the limitations of BMI-based risk assessment, heterogeneity in phenotype definitions, the distinction between established and emerging pathogenic mechanisms, and the implications for cardiometabolic risk assessment and management.

## 2 Epidemiology

The epidemiology of metabolic syndrome (MetS) and related phenotypes in individuals with normal body mass index (BMI 18.5–24.9 kg/m^2^) demonstrates a substantial and clinically significant burden, challenging the adequacy of BMI as a solitary marker of metabolic health [14]. This section details the global prevalence, determinants, and public health implications of MONW phenotypes.

### 2.1 Prevalence estimates and global variation

Prevalence estimates for MONW have varied widely, ranging from approximately 0.9% to 59% in previous global studies [15], [19], [20]. These variations are principally driven by the specific metabolic criteria applied and the demographic characteristics of the study populations. When defined by strict NCEP-ATP III MetS criteria, prevalence in general population samples was generally lower. For instance, In the U.S. NHANES cohort, the prevalence of MetS increased progressively across BMI categories, including within the normal-weight range, indicating that metabolic risk is heterogeneous even among individuals without obesity [20]. Prevalence estimates for MONW and related phenotypes vary substantially across populations, reflecting differences in phenotype definitions, metabolic criteria, BMI thresholds, age, sex, and ethnicity. For instance, MetS was reported in 7.6% of normal-weight adults in a Brazilian urban population [21], 6.4% in Iranian cohort [13]. and 8.6% of normal-weight adults in the United States [8]. These estimates should not be interpreted as directly comparable because the underlying studies differed in population characteristics and diagnostic definitions. Consequently, the true global prevalence of NWMS remains uncertain.

Conversely, prevalence estimates are substantially higher when using definitions of intermediate metabolic risk, such as the presence of ≥2 abnormalities of the following: (1) hypertension (systolic blood pressure ≥130 mmHg and/or diastolic ≥85 mmHg and/or antihypertensive therapy); (2) hypertriglyceridemia (triglycerides ≥1.7 mmol/L); (3) low HDL-C (<1.0 mmol/L in men or <1.3 mmol/L in women); or (4) elevated fasting glucose (≥100 mg/dL or glucose-lowering drug treatment [9]. Applying such criteria, a nationally representative Chinese study found 14% of non-diabetic, normal-weight adults were metabolically unhealthy [9], while a larger community-based study reported 34% had ≥2 metabolic abnormalities, with 16.1% meeting specific MONW criteria [15]. A review of Polish study highlights the extreme of this range, reporting MONW prevalence from 14.7% to 59% across different studies, underscoring the profound impact of methodological heterogeneity [22].

This methodological heterogeneity is compounded by ethnic variations in body composition and cardiometabolic risk thresholds. For instance, South Asian populations are known to have a higher percentage of body fat and greater propensity for visceral adiposity at lower BMIs compared to Caucasians [23]. Consequently, when applying ethnicity-specific cut-offs for waist circumference or body fat percentage, the estimated prevalence of MONW/MUNW in these populations increases substantially, highlighting the inadequacy of universal BMI cut-points for global risk assessment [24], [25].

Despite growing data from the Americas, Asia, and Europe, the epidemiology of MONW remains markedly understudied in large regions of the world. Notably, there is a dearth of population-based data from the African continent, as well as from many parts of South Asia, the Middle East beyond Iran, and Eastern Europe. Preliminary data from sub-Saharan Africa suggest a complex and non-linear relationship between BMI and metabolic disease, with emerging evidence of a notable MONW prevalence in urbanizing populations experiencing rapid nutritional transitions [26]. This gap is critical, as the pathophysiology of adiposity and its metabolic consequences may differ substantially across ethnicities due to genetic, lifestyle, and environmental factors. The lack of data from these regions limits the generalizability of current global prevalence estimates and hinders the development of targeted public health strategies.

Interpretation of these prevalence estimates requires consideration of substantial methodological heterogeneity. Studies variably apply MONW, MUNW, NWMS, or NWO definitions, with differences in the number and combination of metabolic abnormalities required for classification. BMI thresholds also vary across populations, particularly with the use of lower ethnicity-specific cut-offs in Asian populations. Consequently, individuals classified as normal weight or metabolically unhealthy in one study may not meet the corresponding definition in another. These differences in phenotype definition and BMI thresholds likely contribute substantially to the wide variation in reported prevalence and limit direct comparisons across studies and populations.

Accordingly, the reported prevalence range of 0.9–59% should not be interpreted as a single estimate of the global burden of NWMS. Rather, it reflects substantial variation in phenotype definitions, BMI thresholds, metabolic criteria, and population characteristics across studies. Estimates derived using standardized MetS criteria are therefore more clinically interpretable than comparisons across heterogeneous MONW, MUNW, and NWO definitions. At present, the available evidence does not support a precise global prevalence estimate for NWMS, underscoring the need for population-based studies using harmonized definitions and reporting standards.

The geographic distribution of available evidence remains uneven. Most prevalence data originate from Asia, Europe, and North America, whereas evidence from Africa, parts of South Asia, the Middle East, Eastern Europe, and other underrepresented regions remains limited. Consequently, estimates derived from data-rich populations should not be interpreted as representative of the global burden of NWMS. This limitation is particularly important given geographic and ethnic differences in body composition, visceral adiposity, dietary exposures, and cardiometabolic susceptibility. Robust population-based studies using harmonized definitions and standardized metabolic and anthropometric assessments are therefore needed to establish the prevalence and clinical significance of NWMS across diverse populations.

### 2.2 The intra-normal BMI risk gradient

A consistent and clinically important observation is that metabolic risk is heterogeneous within the normal BMI range. Studies indicate that individuals toward the upper end of the normal BMI range may have a greater burden of metabolic abnormalities than those with lower-normal BMI [20]. Among Polish women, for example, those in the second and third tertiles of normal BMI had higher odds of MetS (OR 2.22 and 3.97, respectively) than those in the lowest tertile [14]. These findings indicate that normal BMI does not confer uniform metabolic risk and reinforce the importance of assessing cardiometabolic factors independently of BMI.

### 2.3 Demographic determinants: age and sex disparities

The distribution of the metabolically obese, normal-weight (MONW) phenotype exhibits distinct demographic patterns, primarily shaped by age and sex. Sex-based differences are most pronounced in younger and middle-aged adults. Multiple studies indicate that men under 45 years of age have a significantly higher prevalence of MONW compared to women of the same age group [14], [15]. This disparity is frequently attributed to a greater male propensity for visceral adiposity at a given body mass index (BMI) [14]. Since waist circumference (WC), a key marker of central obesity, is strongly and significantly associated with all metabolically unhealthy states [20], this underlying difference in fat distribution is a critical driver of the observed sex gap [13].

Furthermore, hormonal influences are fundamental to this disparity. In premenopausal women, estrogen is thought to promote a gynoid (subcutaneous) fat distribution and enhance insulin sensitivity, offering relative protection [27]. In contrast, younger men have higher androgen levels which may preferentially promote visceral adipogenesis. The role of sex hormone-binding globulin (SHBG) is also notable, as lower levels common in insulin-resistant states, are independently associated with MONW in both sexes but may manifest earlier in men [28].

This pattern tends to attenuate and often reverse in older age groups. Research shows a lower prevalence of MONW among men compared to women aged 50 years and older [15]. This shift aligns with the well-documented post-menopausal increase in cardiometabolic risk among women, a transition influenced by factors such as menopause itself and changes in body composition [15]. The menopausal transition is characterized by a decline in estrogen and a subsequent shift toward a more android fat distribution, with increased visceral and hepatic fat accumulation even without a change in total weight [29]. This explains not only the convergence of MONW prevalence between older men and women but also the particularly adverse cardiometabolic profile observed in postmenopausal women with the phenotype. Furthermore, the age-related decline in growth hormone and testosterone in men may also alter fat distribution and contribute to the attenuation of the disparity [30].

Beyond age and sex, other determinants modulate MONW risk. Higher BMI or WC, a family history of hypertension, diabetes, or cardiovascular diseases are consistently associated with increased risk [15]. Conversely, higher education levels, regular alcohol consumption, and balanced or vegetarian dietary preferences have been identified as potential protective factors [15].

The clinical significance of the MONW phenotype is underscored by its substantial association with cardiometabolic disease. Individuals with MONW face a 1.5 to 3-fold higher risk of cardiovascular disease (CVD) compared to their metabolically healthy normal-weight counterparts, a risk that sometimes exceeds that of individuals classified as “metabolically healthy obese” [10], [31]. The risk for developing type 2 diabetes is even more pronounced, ranging from 3 to 11-fold higher than in metabolically healthy individuals, with risk escalating alongside each additional component of the metabolic syndrome [10].

### 2.4 Public health significance and burden

The epidemiological profile of MUNW underscores a significant public health challenge. While the prevalence of full MetS in normal-weight populations may appear modest in some studies (e.g., 8.6% in the US [8]) the far larger reservoir of individuals with pre-MetS or intermediate metabolic abnormalities, constituting up to a third of some normal-weight populations [9], represents a vast, high-risk group that is systematically missed by BMI-centric screening. These individuals remain at elevated risk for incident type 2 diabetes and cardiovascular disease [9]. The observed burden, intra-BMI risk gradient, and demographic patterning of MUNW support metabolic risk assessment beyond BMI alone, particularly in normal-weight individuals with central adiposity or other cardiometabolic risk factors.

## 3 Clinical presentation and diagnostic criteria

The clinical presentation of metabolic syndrome (MetS) in normal-weight individuals mirrors its classical form but is characterized by a critical paradox: the presence of significant cardiometabolic dysfunction in the absence of generalized obesity.

### 3.1 Clinical manifestations and signs

Patients with normal-weight MetS are often asymptomatic in the early stages, making detection reliant on systematic screening. The most consistent and often sole visible sign is central adiposity, evidenced by an elevated waist circumference despite a normal body mass index (BMI 18.5–24.9 kg/m^2^). This distinguishes the phenotype from generalized obesity, where excess adiposity is more evenly distributed. As insulin resistance progresses, subtle clinical signs may emerge. Cutaneous manifestations such as acanthosis nigricans (velvety, hyperpigmented plaques in intertriginous areas) and multiple skin tags (acrochordons) are common markers of hyperinsulinemia frequently observed in this population [32]. Non-specific symptoms of compensatory hyperinsulinemia and hyperglycemia, including fatigue, brain fog, or increased hunger, may be reported. With progressive β-cell dysfunction, symptoms of overt hyperglycemia, such as polyuria, polydipsia, and blurred vision can develop. A proportion of affected individuals may also have conditions associated with insulin resistance, including metabolic dysfunction-associated steatotic liver disease (MASLD), polycystic ovary syndrome (PCOS), and hyperuricaemia/gout; the presence of these conditions in a normal-weight individual may warrant further assessment of cardiometabolic risk [11], [12]. The presence of these conditions in a normal-weight individual should prompt a comprehensive metabolic evaluation. From a clinical assessment perspective, suspicion should be heightened by specific findings such as a family history of premature cardiovascular disease or type 2 diabetes, a personal history of gestational diabetes or PCOS, and the presence of acanthosis nigricans or skin tags [32]. Laboratory clues beyond standard MetS components can include elevated liver enzymes, which may prompt further evaluation for MASLD, high-sensitivity C-reactive protein (hs-CRP), or an elevated homeostasis model assessment of insulin resistance (HOMA-IR) index, even in the setting of normal fasting glucose [19].

### 3.2 Diagnostic criteria and frameworks

Diagnosis formally hinges on established criteria for metabolic syndrome. The most commonly applied are the harmonized criteria, requiring the presence of at least three of five components [9], [33]: (1) elevated waist circumference (using population- and country-specific cutoffs); (2) elevated triglycerides (≥150 mg/dL or 1.7 mmol/L) or drug treatment for hypertriglyceridemia; (3) reduced HDL cholesterol (<40 mg/dL in men, <50 mg/dL in women) or drug treatment for low HDL-C; (4) elevated blood pressure (systolic ≥130 and/or diastolic ≥85 mmHg) or antihypertensive drug treatment; and (5) elevated fasting glucose (≥100 mg/dL) or drug treatment for hyperglycemia. For the specific diagnosis of metabolically obese, normal weight (MONW), these criteria are applied specifically to individuals within the normal BMI range. This operational definition is clinically pragmatic but has limitations, as it may miss individuals with fewer than three criteria who are still at high risk (so-called “pre-MetS").

### 3.3 Limitations of BMI in identifying metabolic risk

BMI does not distinguish fat from lean mass or adequately characterize regional adipose tissue distribution and therefore has important limitations for cardiometabolic risk assessment. Normal-weight obesity (NWO), characterized by excess body-fat percentage despite normal BMI, illustrates this limitation [5]. Although NWO may overlap with NWMS, the two phenotypes are not synonymous because increased body-fat percentage does not necessarily indicate the presence of metabolic syndrome.

Additional anthropometric, body-composition, and metabolic assessments may provide supportive phenotypic information in selected individuals. Waist circumference and waist-to-height ratio provide accessible measures of central adiposity [34], while bioelectrical impedance analysis (BIA) and dual-energy X-ray absorptiometry (DXA) can provide additional information on fat and lean mass [35]. Computed tomography (CT) and magnetic resonance imaging (MRI) permit more detailed assessment of visceral and ectopic adiposity but are generally less suitable for routine screening because of cost, accessibility, and, for CT, radiation exposure [36], [37].

### 3.4 Body composition and adiposity assessment

Because BMI does not capture body-fat distribution or distinguish adipose from lean tissue, complementary measures may improve cardiometabolic risk assessment in normal-weight individuals [17], [37]. Waist circumference and waist-to-height ratio are inexpensive and clinically accessible measures of central adiposity, whereas bioelectrical impedance analysis and dual-energy X-ray absorptiometry provide additional information on fat and lean mass [35]. Computed tomography and magnetic resonance imaging offer more precise assessment of visceral and ectopic adiposity but are limited by cost and accessibility [38]. A pragmatic approach is therefore to complement BMI with simple anthropometric measures in routine practice and reserve advanced body-composition assessment or imaging for selected individuals. However, the cost-effectiveness of these advanced modalities for routine NWMS screening remains uncertain.

### 3.5 Cardiovascular risk stratification

Normal BMI should not be equated with low cardiovascular risk when metabolic abnormalities are present. Cardiovascular risk assessment in NWMS should integrate established risk factors, including blood pressure, glycaemic status, lipid profile, smoking, and family history. In selected individuals with uncertain risk, coronary artery calcium scoring may provide additional risk stratification and identify subclinical atherosclerosis [39]. Metabolic dysfunction-associated steatotic liver disease may further identify individuals with an adverse cardiometabolic profile and increased cardiovascular risk [40], [41].

Importantly, cardiometabolic risk at a given BMI varies across ethnic populations. South Asian populations exhibit greater visceral and ectopic adiposity and may develop insulin resistance, type 2 diabetes, and cardiovascular disease at lower BMI levels than many European-origin populations [42]. These differences reinforce the limitations of universal BMI thresholds and support a multidimensional approach integrating metabolic abnormalities, central adiposity, and, when clinically appropriate, assessment of subclinical cardiovascular disease.

### 3.6 Screening and differential diagnostic approach

Recognition of NWMS requires both identification of metabolic abnormalities and exclusion of related phenotypes or secondary causes. Metabolically healthy obesity (MHO) describes individuals with obesity but without major metabolic abnormalities, although metabolic health may deteriorate over time [43], [44]. Sarcopenic obesity is characterized by the coexistence of excess adiposity and reduced muscle mass or function and is particularly relevant in older adults [45]. Normal-weight obesity should also be distinguished from NWMS because increased body-fat percentage may occur without meeting diagnostic criteria for metabolic syndrome. Secondary endocrine disorders, including Cushing syndrome, hypothyroidism, and growth hormone deficiency, should be investigated when suggested by the clinical presentation.

Rather than universal screening of all normal-weight adults, a risk-based approach is more appropriate. Assessment should be prioritized in individuals with central adiposity, family history of type 2 diabetes or premature cardiovascular disease, previous gestational diabetes, polycystic ovary syndrome, physical inactivity, or other cardiometabolic risk factors. Initial evaluation can incorporate waist circumference or waist-to-height ratio, blood pressure, fasting glucose or HbA1c, and lipid profile. Abnormal findings may prompt more detailed metabolic or body-composition assessment, with advanced imaging reserved for selected clinical or research settings. A proposed tiered approach integrating basic screening, enhanced phenotyping, and advanced characterization is summarized in Table 1.

**Table 1:** Proposed tiered diagnostic approach to suspected normal-weight metabolic syndrome.

Assessment Tier	Core Metrics	Supportive/Advanced Metrics	Purpose
Tier 1: Basic Screening (Primary Care)	BMI, waist circumference or waist-to-height ratio, blood pressure, fasting glucose or HbA1c, fasting lipid profile	-	Identify metabolic abnormalities and determine whether established MetS criteria are fulfilled despite normal BMI.
Tier 2: Enhanced Phenotyping (Increased Risk/Preventive Clinic)	Tier 1 assessments + body-composition assessment (BIA or DXA, where available)	OGTT, selected measures of insulin resistance (e.g., HOMA-IR), hs-CRP, liver biochemistry, and assessment for MASLD when clinically indicated	Characterize adiposity and metabolic dysfunction not adequately captured by BMI and identify associated metabolic disease.
Tier 3: Advanced Characterization (Research/Specialist)	Detailed body-composition and metabolic phenotyping	MRI or CT assessment of visceral/ectopic fat; selected adipokine, cytokine, or other mechanistic biomarkers	Characterize complex phenotypes and investigate visceral, ectopic, and molecular abnormalities

**Abbreviations**: BIA, bioelectrical impedance analysis; BMI, body mass index; CT, computed tomography; DXA, dual-energy X-ray absorptiometry; HbA1c, glycated haemoglobin; HOMA-IR, Homeostatic Model Assessment of Insulin Resistance; hs-CRP, high-sensitivity C-reactive protein; MASLD, metabolic dysfunction-associated steatotic liver disease; MetS, metabolic syndrome; MRI, magnetic resonance imaging; OGTT, oral glucose tolerance test.

## 4 Pathogenic mechanisms

The pathophysiology of metabolic dysfunction in normal-weight individuals is multifactorial and remains incompletely characterized. Current evidence most consistently implicates disproportionate visceral adiposity, ectopic lipid deposition, insulin resistance, adipose tissue dysfunction, and chronic low-grade inflammation as central pathogenic mechanisms [17], [18], [46]. These processes are closely interconnected and supported by human metabolic and imaging studies; however, much of the molecular evidence derives from broader populations with obesity, insulin resistance, or metabolic syndrome rather than specifically defined normal-weight phenotypes. Gut microbiota dysbiosis, oxidative stress, ferroptosis, and genetic and epigenetic susceptibility may represent additional contributors, but their phenotype-specific roles remain less well established. This distinction is important when interpreting mechanistic evidence and differentiating established pathogenic pathways from emerging hypotheses.

### 4.1 Visceral adiposity and ectopic fat deposition

In the MONW phenotype, disproportionate visceral adipose tissue (VAT) accumulation is a defining feature, even in the absence of elevated total body mass. VAT differs from subcutaneous fat in several critical metabolic and endocrinologic properties. It has a higher basal lipolytic rate, releasing free fatty acids (FFAs) directly into the portal circulation, thereby exposing the liver to a continuous flux of lipids [43], [44]. This portal FFA overflow induces hepatocellular stress by activating protein kinase C (PKC) isoforms, which serine-phosphorylate insulin receptor substrate-1 (IRS-1) and inhibit insulin-dependent activation of PI3K/AKT signaling. Functionally, this reduces suppression of gluconeogenesis and enhances hepatic VLDL-triglyceride secretion, driving systemic hyperglycemia and hypertriglyceridemia. Concurrently, chronic lipid overload induces endoplasmic reticulum (ER) stress, mitochondrial dysfunction, and oxidative stress, triggering hepatocyte apoptosis and secretion of pro-inflammatory mediators such as IL-6 and TNF-α, which propagate systemic insulin resistance [18], [47].

Skeletal muscle is similarly affected. In MONW individuals, intramyocellular lipid (IMCL) accumulation generates bioactive lipid intermediates, including diacylglycerols (DAGs) and ceramides, which activate novel PKC isoforms (PKCθ in muscle). These kinases phosphorylate IRS-1 on serine residues, disrupting insulin receptor signaling, impairing GLUT4 translocation, and reducing glucose uptake into myocytes (Fig. 1) [46]. This mechanism contributes to profound peripheral insulin resistance, which is a hallmark of MONW, despite normal body weight.

**Fig. 1:**
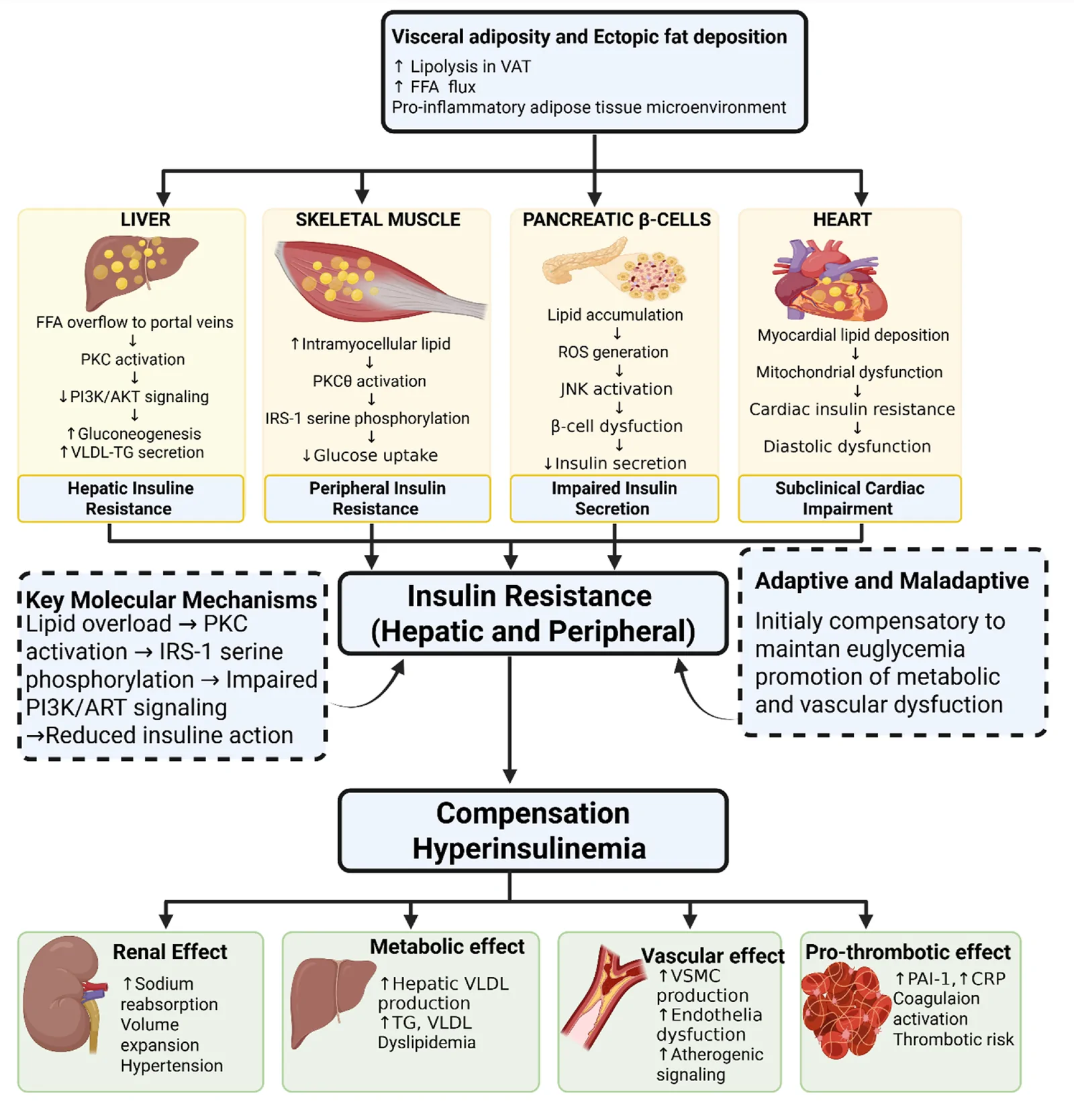
Pathogenic mechanisms of normal-weight metabolic syndrome. Visceral adiposity and ectopic lipid deposition increase free fatty acid (FFA) flux and promote a pro-inflammatory adipose tissue milieu, contributing to hepatic and skeletal-muscle insulin resistance, pancreatic β-cell dysfunction, and myocardial metabolic impairment. These tissue-specific abnormalities converge on systemic insulin resistance and compensatory hyperinsulinemia. Persistent insulin resistance and hyperinsulinemia may subsequently promote renal sodium retention and hypertension, hepatic very-low-density lipoprotein (VLDL) overproduction and dyslipidemia, vascular dysfunction, and a prothrombotic state, collectively increasing cardiometabolic risk. The pathways shown represent an integrated mechanistic framework; several molecular mechanisms have been extrapolated from broader metabolic-disease literature and are not specific to normal-weight metabolic syndrome. FFA, free fatty acid; IRS-1, insulin receptor substrate-1; JNK, c-Jun N-terminal kinase; PAI-1, plasminogen activator inhibitor-1; PI3K/AKT, phosphoinositide 3-kinase/protein kinase B; PKC, protein kinase C; ROS, reactive oxygen species; TG, triglycerides; VAT, visceral adipose tissue; VLDL, very-low-density lipoprotein; VSMC, vascular smooth muscle cell.

Ectopic fat deposition in the pancreas further exacerbates metabolic dysfunction. Lipid infiltration in β-cells induces lipotoxic stress, generating ROS and activating JNK-mediated signaling pathways that impair insulin secretion [47]. In the heart, myocardial lipid accumulation promotes cardiac insulin resistance, disrupts mitochondrial function, and contributes to diastolic dysfunction, predisposing even normal-weight individuals to subclinical cardiac impairment. These processes demonstrate that the distribution and activity of adipose tissue, rather than total mass, dictate metabolic health.

### 4.2 Insulin resistance and hyperinsulinemia

In the MONW phenotype, insulin resistance emerges from the integrated effects of visceral and ectopic lipid accumulation, adipose tissue dysfunction, and chronic low-grade inflammation [48], [49]. In the liver, lipid-induced activation of protein kinase C (PKC) isoforms promotes serine phosphorylation of insulin receptor substrate-1 (IRS-1), impairing downstream PI3K/AKT signaling, reducing glycogen synthesis, and blunting insulin-mediated suppression of hepatic gluconeogenesis [50], [51]. Skeletal muscle is similarly compromised: accumulation of diacylglycerols and ceramides disrupts IRS-1 function, attenuates GLUT4 translocation, and diminishes glucose uptake, establishing profound peripheral insulin resistance [52]. To maintain euglycemia, pancreatic β-cells augment insulin secretion, resulting in compensatory hyperinsulinemia [46]. While initially adaptive, chronic hyperinsulinemia exerts pleiotropic metabolic and vascular effects: it enhances renal sodium reabsorption, promoting hypertension; stimulates hepatic VLDL production, exacerbating dyslipidemia; and drives vascular smooth muscle proliferation and endothelial dysfunction, accelerating atherogenesis (Fig. 1) [43], [50].

### 4.3 Adipose tissue dysfunction and chronic inflammation

Hypertrophic VAT is not only a site of lipid overflow but also a chronic inflammatory microenvironment. Stressed adipocytes release chemokines (MCP-1, CCL2) that recruit immune cells, primarily macrophages. These macrophages form crown-like structures (CLSs) around necrotic or apoptotic adipocytes, amplifying local inflammation [18]. Dysregulated adipocytes and macrophages secrete elevated levels of pro-inflammatory adipokines, including TNF-α, IL-6, resistin, and leptin. TNF-α and IL-6 activate stress kinases (JNK, IKKβ) that phosphorylate IRS-1 on serine residues, impairing downstream PI3K/AKT signaling and systemic insulin action [47].

Concurrently, the production of anti-inflammatory adipokines, particularly adiponectin, is suppressed. Adiponectin normally promotes insulin sensitivity via AMPK and PPARα pathways and exerts anti-atherogenic effects [53]. The imbalance between elevated pro-inflammatory mediators and reduced adiponectin establishes a pro-inflammatory, pro-thrombotic systemic milieu, reflected by increased circulating C-reactive protein (CRP) and plasminogen activator inhibitor-1 (PAI-1) [54]. This environment fosters endothelial dysfunction, coagulation activation, and accelerates atherogenesis, linking VAT dysfunction to cardiometabolic disease, independent of BMI.

### 4.4 Sex hormones and metabolic susceptibility

Sex differences in NWMS may partly reflect hormonal regulation of adipose tissue distribution and metabolic function. Estrogen signaling generally favors subcutaneous rather than visceral fat deposition and contributes to insulin sensitivity, adipocyte function, and regulation of inflammatory and lipid metabolic pathways [55]. Declining estrogen levels during menopause are associated with redistribution of fat toward visceral depots, impaired insulin sensitivity, and a less favorable cardiometabolic profile, potentially increasing susceptibility to metabolic dysfunction despite relatively stable BMI [56]. Sex-specific differences in skeletal muscle, adipokine signaling, and hepatic lipid metabolism may further modify metabolic risk. However, direct mechanistic evidence specifically in NWMS remains limited, and much of the current understanding is extrapolated from broader studies of menopause, adiposity, and metabolic disease.

### 4.5 Emerging and putative pathogenic mechanisms

#### 4.5.1 Gut microbiota dysbiosis

Gut microbiota dysbiosis may contribute to metabolic dysfunction through disruption of the intestinal–metabolic axis, although direct mechanistic evidence in normal-weight metabolic syndrome remains limited. Alterations in microbial community structure and function can compromise intestinal epithelial integrity and increase systemic exposure to microbial products, particularly lipopolysaccharide, thereby activating innate immune pathways and sustaining low-grade inflammation [57], [58]. In parallel, dysbiosis may alter the production of short-chain fatty acids, bile-acid derivatives, and other microbiota-derived metabolites involved in intestinal barrier homeostasis, glucose and lipid metabolism, and host immune regulation [58], [59], [60]. These perturbations can converge on inflammatory and metabolic signaling pathways that impair insulin action in the liver, skeletal muscle, and adipose tissue, potentially promoting insulin resistance despite the absence of generalized obesity.

This mechanism is particularly relevant to the normal-weight phenotype because metabolic dysfunction may reflect qualitative abnormalities in adipose tissue and host–microbiome interactions rather than excess body mass alone. Nevertheless, most supporting evidence derives from observational studies and experimental models of obesity, insulin resistance, or metabolic syndrome, and microbial signatures vary substantially with diet, medications, ethnicity, geography, and analytical methods [59], [60]. Thus, gut dysbiosis represents a biologically plausible but still emerging mechanism in normal-weight metabolic syndrome; longitudinal and mechanistic studies in rigorously phenotyped normal-weight populations are required to establish causality and determine whether microbiome alterations are distinct from those observed in obesity-associated metabolic syndrome.

#### 4.5.2 Oxidative stress and ferroptosis

Oxidative stress may amplify metabolic dysfunction by disrupting the balance between reactive oxygen species generation and antioxidant defenses. Excess reactive oxygen species can impair mitochondrial function, promote lipid peroxidation, and perturb insulin-signaling pathways, thereby contributing to insulin resistance and cellular metabolic stress [61]. Although these processes are well recognized across metabolic disorders, their independent contribution to metabolic dysfunction in normal-weight individuals remains insufficiently characterized.

Ferroptosis represents a potential mechanistic extension of this redox disturbance. This iron-dependent form of regulated cell death occurs when iron-driven phospholipid peroxidation overwhelms cellular antioxidant defenses, resulting in oxidative membrane damage and loss of cellular integrity [62]. Metabolic disturbances in lipid handling, iron homeostasis, and antioxidant capacity may increase susceptibility to ferroptotic injury, providing a plausible link between metabolic stress and tissue dysfunction [63], [64]. However, evidence for ferroptosis in NWMS remains indirect, with current support derived predominantly from experimental models and studies of metabolic liver disease rather than specifically characterized normal-weight populations. Whether ferroptosis contributes independently to NWMS or represents a pathway shared with obesity-associated metabolic syndrome therefore remains unresolved. Accordingly, ferroptosis should be regarded as an emerging mechanistic hypothesis rather than an established feature of NWMS, warranting direct investigation in rigorously phenotyped normal-weight populations.

### 4.6 Genetic and environmental determinants

The MONW phenotype arises from a complex interplay between genetic predisposition and modifiable lifestyle factors. Specific genetic polymorphisms have been identified as key contributors to metabolic dysfunction in normal-weight individuals, even in the absence of excess adiposity. Variants in genes such as CDKAL1, which regulates insulin secretion through its role as a CDK5 regulatory subunit-associated protein, and GCKR, which governs glucokinase regulatory activity and thereby influences glucose and lipid metabolism, have been consistently associated with metabolic disorders in individuals maintaining a normal BMI [65], [66]. These polymorphisms suggest that metabolic dysregulation can be deeply rooted in an individual's genetic architecture, making body weight an insufficient and potentially misleading indicator of true metabolic health. The polygenic nature of the MONW phenotype further implies that risk stratification must extend beyond anthropometric measures to incorporate genomic profiling, particularly in populations with a family history of type 2 diabetes, dyslipidemia, or cardiovascular disease.

Environmental factors serve as critical triggers that unmask metabolic vulnerability in genetically susceptible individuals, effectively converting a latent predisposition into a clinically manifest phenotype. A sedentary lifestyle is among the most significant modifiable risk factors, as physical inactivity reduces insulin sensitivity and impairs lipid oxidation, promoting ectopic fat deposition in visceral and hepatic compartments despite the absence of overall weight gain [25], [45]. Dietary patterns characterized by high intake of saturated fats and refined carbohydrates further exacerbate this process by driving postprandial dyslipidemia, hepatic lipogenesis, and systemic insulin resistance. Additionally, smoking induces oxidative stress and promotes visceral fat redistribution, while moderate alcohol consumption contributes to hepatic lipid accumulation and altered adipokine signaling. Therefore, these environmental determinants highlight that the MONW phenotype is not merely a genetic inevitability but rather a largely preventable condition, provided that lifestyle interventions are implemented early and targeted toward individuals identified as genetically at risk.

### 4.7 Psychological and neuroendocrine factors

Psychological stress may contribute to metabolic dysfunction through dysregulation of the hypothalamic–pituitary–adrenal axis and cortisol signaling, potentially promoting insulin resistance, visceral adiposity, and related metabolic abnormalities [67], [68]. Chronic stress may also interact with behavioral factors, including sleep, diet, and physical activity, to amplify cardiometabolic risk. However, direct evidence specifically linking psychological stress to NWMS remains limited; its role should therefore be considered contributory rather than an established phenotype-specific mechanism.

## 5 Management and therapeutic implications

The management of normal-weight MetS necessitates a shift from weight-centric to metabolism-centric care, focusing on improving body composition and metabolic health.

### 5.1 Lifestyle and dietary management

Lifestyle modification is central to the management of NWMS. Regular physical activity, integrating both structured aerobic exercise and resistance training, is paramount. This regimen directly enhances insulin sensitivity in skeletal muscle through improved glucose transporter type 4 (GLUT4) translocation and mitochondrial function, while simultaneously promoting favorable fat redistribution away from visceral and ectopic sites [69]. Critically, these cardiometabolic benefits, including improved blood pressure and lipid profiles, can be achieved independently of significant changes in total body weight [64], [65]. A complementary and equally critical nutritional strategy must explicitly target the reduction of metabolically deleterious fat. An evidence-based approach emphasizes a high-fiber, low-glycemic index dietary pattern, such as the Mediterranean diet, which is rich in phytochemicals, quality protein, and healthy monounsaturated and polyunsaturated fats [70]. This strategy is fundamentally designed to displace pro-inflammatory dietary components by strictly limiting refined carbohydrates, added sugars, and saturated fats, thereby directly improving hepatic and peripheral insulin signaling.

In NWMS, lifestyle management should prioritize improvement in body composition and metabolic fitness rather than weight loss alone. This is particularly important for individuals at the lower end of the normal BMI range (18.5–21.0 kg/m^2^), in whom further weight reduction may compromise lean mass. Resistance training should complement aerobic exercise because preservation or augmentation of skeletal muscle can improve insulin sensitivity and glucose disposal [71]. Adequate protein intake and overall dietary quality are also important for maintaining muscle mass, particularly in older adults and individuals at risk of sarcopenia [72]. Lifestyle interventions should therefore be individualized according to baseline BMI, body composition, and metabolic abnormalities, emphasizing reduction of central adiposity while preserving or increasing skeletal muscle.

### 5.2 Pharmacological management

Pharmacological management of NWMS should target established cardiometabolic abnormalities rather than body weight itself, as no pharmacological agent is specifically approved for NWMS. Treatment of dysglycaemia, hypertension, and atherogenic dyslipidaemia should therefore follow established disease-specific guidelines [73].

Metformin may be considered when clinically indicated for dysglycaemia or diabetes, primarily through its effects on hepatic glucose production and insulin sensitivity [74]. Statins and antihypertensive therapies should similarly be prescribed according to established cardiovascular-risk and disease-specific indications rather than NWMS status alone.

Newer glucose-lowering therapies may provide additional cardiometabolic benefits in appropriately selected patients. GLP-1 receptor agonists improve glycaemic control and cardiovascular outcomes, while SGLT2 inhibitors provide cardiovascular and kidney protection in patients with relevant established indications, including type 2 diabetes, heart failure, or chronic kidney disease [84]. Dual GIP/GLP-1 receptor agonists, particularly tirzepatide, provide substantial improvements in glycaemic and metabolic parameters [75]. However, these therapies have not been adequately evaluated specifically in NWMS and should not currently be prescribed solely on the basis of normal-weight metabolic dysfunction.

### 5.3 Emerging and investigational therapies

Several mechanistically targeted interventions remain investigational. Modulation of the gut microbiome through dietary interventions, prebiotics, probiotics, or other microbiota-directed strategies has been proposed as a means of modifying metabolic inflammation [76]. Similarly, anti-inflammatory approaches targeting chronic low-grade inflammation are under investigation [77]. Ferroptosis and related oxidative cell-death pathways represent additional potential therapeutic targets; however, evidence remains predominantly experimental and is not specific to NWMS. These approaches should therefore be regarded as hypothesis-generating rather than established therapeutic strategies.

### 5.4 Evidence base and therapeutic limitations

Evidence for the management of NWMS remains limited. Most recommendations regarding dietary modification, physical activity, resistance training, and pharmacological therapy are extrapolated from studies of obesity, type 2 diabetes, or metabolic syndrome rather than randomized trials specifically enrolling individuals with NWMS/MONW [35]. Consequently, although these interventions target relevant metabolic abnormalities, their magnitude of benefit and optimal implementation in normal-weight populations remain uncertain.

Pharmacological therapy should therefore be guided by established indications for individual cardiometabolic conditions rather than NWMS status alone [78]. Dedicated randomized trials are needed to determine whether interventions targeting visceral adiposity, insulin resistance, or skeletal muscle provide phenotype-specific benefit in NWMS.

## 6 Conclusion

Normal-weight metabolic syndrome is a clinically important phenotype characterized by cardiometabolic abnormalities despite normal BMI. Its pathogenesis appears to involve visceral and ectopic adiposity, insulin resistance, adipose tissue dysfunction, and chronic low-grade inflammation, whereas the roles of gut microbiota dysbiosis, ferroptosis, and other emerging pathways remain less established. Recognition of metabolic risk beyond BMI is therefore important for identifying susceptible individuals.

Future research should prioritize harmonized diagnostic definitions, validation of risk-based screening approaches, and clinical trials specifically involving normal-weight populations. Such evidence is needed to clarify the global burden of NWMS and inform phenotype-specific prevention and management strategies.

## CRediT authorship contribution statement

**John Nzobokela:** Writing – review & editing, Writing – original draft, Visualization, Validation, Project administration, Methodology, Investigation, Conceptualization. **Bisom Chikwanka Mweene:** Writing – review & editing, Writing – original draft, Visualization, Validation, Methodology, Conceptualization. **Elletina Nchimunya Sinamwenda:** Writing – review & editing, Writing – original draft, Visualization, Validation, Conceptualization. **Situmbeko Liweleya:** Writing – review & editing, Writing – original draft, Visualization, Validation, Conceptualization. **Sepiso K. Masenga:** Writing – review & editing, Writing – original draft, Visualization, Validation, Conceptualization. **Lweendo Muchaili:** Writing – review & editing, Writing – original draft, Visualization, Validation, Supervision, Project administration, Methodology, Investigation, Data curation, Conceptualization. **Benson M. Hamooya:** Writing – review & editing, Writing – original draft, Visualization, Validation, Supervision, Project administration, Methodology, Data curation, Conceptualization.

## Funding

This study did not receive funding.

## Conflict of interest

The authors declare that the research was conducted in the absence of any commercial or financial relationships that could be construed as a potential conflict of interest.
